# The *in vitro* toxicology of Swedish snus

**DOI:** 10.3109/10408444.2012.666660

**Published:** 2012-03-09

**Authors:** Christopher R. E. Coggins, Mark Ballantyne, Margareta Curvall, Lars-Erik Rutqvist

**Affiliations:** 1 Carson Watts Consulting, King, NC, USA; 2 Covance Laboratories Ltd., Harrogate, UK; 3 Swedish Match AB, Stockholm, Sweden

**Keywords:** Swedish snus, smokeless tobacco, genotoxicity, cytotoxicity, positive controls, water extraction, DMSO extraction

## Abstract

Three commercial brands of Swedish snus (SWS), an experimental SWS, and the 2S3 reference moist snuff were each tested in four *in vitro* toxicology assays. These assays were: *Salmonella* reverse mutation, mouse lymphoma, *in vitro* micronucleus, and cytotoxicity. Water extractions of each of the 5 products were tested using several different concentrations; the experimental SWS was also extracted using dimethyl sulfoxide (DMSO). Extraction procedures were verified by nicotine determinations. Results for SWS in the mutagenicity assays were broadly negative: there were occasional positive responses, but these were effectively at the highest concentration only (concentrations well above those suggested by regulatory guidelines), and were often associated with cytotoxicity. The 2S3 reference was unequivocally positive in one of the three conditions of the micronucleus assay (MNA), at the highest concentration only. Positive controls produced the expected responses in each assay. The SWS data are contrasted with data reported for combusted tobacco in the form of cigarettes, where strongly positive responses have been routinely reported for mutagenicity and cytotoxicity. These negative findings in a laboratory setting concur with the large amount of epidemiological data from Sweden, data showing that SWS are associated with considerably lower carcinogenic potential when compared with cigarettes.

## Introduction

Swedish snus (SWS) are moist to semi-moist, ground, oral tobacco products (Rutqvist et al., 2011), which are often placed in “tea-bag” style packaging which consumers usually place behind their upper lip. The products are made from mainly air-cured tobaccos, water, salt, and flavor additives; they are “pasteurized” in a proprietary heat treatment process which satisfies the hygienic requirements of the Swedish Food Act (Rutqvist et al., 2011). Data from Swedish and US epidemiology studies (Lee and Hamling, 2009; Colilla, 2010) have shown that the use of SWS and other “moist snuff” products have effectively no effect on a broad range of cancers and other diseases (Lee, 2011), a finding in direct contrast with findings repeatedly reported elsewhere for combusted tobacco products such as cigarettes (IARC, 2004; US Department of Health and Human Services, 2004).

Epidemiology data have also shown that SWS has been used by many smokers as an aid to quit smoking, with snus use being associated with an increased probability of being a former smoker (Lund et al., 2011). The efficacy of SWS in smoking cessation was recently demonstrated in two recent (but quite small) clinical trials (Fagerström et al., 2011; Joksic et al., 2011). SWS are very different from smokeless tobacco products (STP) used in regions other than Scandinavia and North America, for example the Sudanese “toombak” (Idris et al., 1998). Therefore, we agree that “*oral tobacco products should not be categorized together when considering the public health implications of their use”* (Stanfill et al., 2011).

Genetic toxicology testing, the study of chemical, physical or biological agents that may interact with DNA resulting in genetic changes, has been a widely used procedure for several decades (Clive et al., 1972; Ames et al., 1973), the active principle being that short-term tests involving genetic changes may be indicative of carcinogenic risk in laboratory animals and in humans. The tests are primarily used on individual compounds (e.g. a novel pharmaceutical), largely as an early stage safety evaluation (if the test produces a positive finding, then there probably will be no further work performed on that compound). It is usual to conduct a complementary battery of tests for each compound and to include tests in both bacterial and mammalian cells. A typical approach would be to use the bacterial (usually *Salmonella typhimurium*) reverse-mutation or “Ames” assay (Ames et al., 1973; Ames et al., 1975; McCann et al., 1975), with additional tests for chromosome damage and aneuploidy (Kirkland et al., 2005; Kirkland et al., 2011). Data on these latter endpoints are often obtained using the micronucleus assay (MNA) and the mouse lymphoma assay (MLA); there are recent suggestions that the only tests needed are *S. typhimurium* and *in vitro* micronucleus (Pfuhler et al., 2007; Kirkland et al., 2011). There are well-established international guidelines on how to perform each of these assays (OECD, 1997a,b,c), including the suggestion that “*wherever possible, the use of an aqueous solvent/vehicle be considered first”*

A related *in vitro* test that is commonly used in combination with the above assays is the neutral red uptake (NRU) assay (Nemes et al., 1979), a test for cytotoxic-ity (Borenfreund and Puerner, 1985). Cytotoxicity is a major concern in genotoxicity assays (dead cells cannot mutate) and this factor has the potential to confound the interpretation of negative results. Compared to the assays listed above, only limited guidelines are available on the NRU assay (Andreoli et al., 2003; King and Jones, 2003).

Combusted tobacco in the form of cigarette smoke condensate (CSC) has been shown to be highly active in a number of *in vitro* toxicology systems (DeMarini, 2004; DeMarini et al., 2008), particularly in the *Salmonella* mutagenicity test for the TA98 and TA100 strains in the presence of the Aroclor^™^ 1254 induced mammalian (rat) liver post-mitochondrial fraction (S9) (Rickert et al., 2007; Patskan et al., 2008; Gaworski et al., 2010). The MLA has also been used with CSC, and again the mixture has been found to be highly mutagenic (Schramke et al., 2006; DeMarini et al., 2008; Werley et al., 2008). An *in vitro* MNA for use with Chinese hamster fibroblast cells has been developed to use whole smoke and different components of this complex mixture (Okuwa et al., 2010). Both particulate and vapor phases of smoke from a reference cigarette generated significant responses in this assay. The NRU assay has also been used routinely in the comparative evaluations of both the gas-vapor and particulate phases of cigarette smoke (Patskan et al., 2008; Gaworski et al., 2010).

The present study was performed to determine whether SWS are active in the *in vitro* toxicology assays classically used to predict carcinogenicity in humans. Although there are published reports on the *in vitro* toxicology of SWS (Jansson et al., 1991), the products evaluated and the tests available in a publication from 20 years ago are quite different from those available today. In particular, the MLA does not appear to have been used previously with SWS. Also, concentrations of tobacco-specific nitrosamines, considered by some to be very important in STP (Österdahl et al., 2004; IARC, 2007; Scientific Committee on Emerging and Newly Identified Health Risks, 2008; World Health Organization, 2008; Stanfill et al., 2011), are now very much lower in SWS than they were 20 years ago, probably the result of altered manufacturing processes (Rutqvist et al., 2011).

## Materials and methods

### SWS and control samples

The following commercial SWS (manufactured by Swedish Match, Stockholm, Sweden) were tested: *General Original Portion Large* (hereafter abbreviated as “G”), *Catch White Portion Large, Licorice* (“CPS”), and *Catch Dry White Portion Mini, Licorice* (“CDM”). An experimental SWS similar to CDM, but with experimental flavoring agents, was also tested (hereafter abbreviated as “CDM2”). The 2S3 reference moist snuff (North Carolina Agricultural Research Service, 2006; Borgerding et al., 2009; Johnson et al., 2009) was also included. Table 1 provides results of chemical analyses of the 5 different test materials, and compares these with the GothiaTek® standard (Rutqvist et al., 2011).

**Table 1:** Characteristics of the Swedish snus and the reference product, compared with the GothiaTek standard.

Parameter	Units	G*	CPS*	CDM*	CDM2*	2S3**	GothiaTek†
Serving size	g	1.0	1.0	0.3	0.3		
Water	%	46.1	48.2	24.0	23.5	54.7	
Sodium chloride	%	9.6	13.1	12.1	10.0	7.32	
pH	units	8.4	8.4	7.5	7.2	7.42	
Nitrite	ppm	2	1	1	2	11.2	7.0
Nicotine	%	1.5	1.6	2.1	2.1	1.38	
NNN^a^	ppm	1.1	1.2	0.9	0.8	3.50	
NNK^b^	ppm	0.3	0.4	0.2	0.2	0.91	
NAT^c^	ppm	0.8	0.8	0.6	0.6	2.35	
NAB^d^	ppm	<0.1	<0.1	<0.1	<0.1	0.18	
TSNA^e^	ppm	2.2	2.4	1.7	1.6	6.95	10.0
Arsenic	ppm	0.14	0.14	0.14	0.11	0.20	0.5
Cadmium	ppm	0.48	0.48	0.57	0.60	1.56	1.0
Chromium	ppm	0.80	1.10	1.60	0.92	1.31	3.0
Lead	ppm	0.32	0.33	0.30	0.27	0.33	2.0
Nickel	ppm	1.2	1.4	2.0	1.6	1.88	
B[a]P^f^	ppb	1.9	1.9	1.3	1.3	79	20

* Data from unpublished Swedish Match analyses. See text for explanation of these abbreviations.

** Data from Borgerding et al. 2009.

† Data from Rutqvist et al. 2011.

a N-nitrosonornicotine.

b 4-(methylnitrosamino)-1-(3-pyridyl)-1-butanone.

c N-nitrosoanatabine.

d N-nitrosoanabasine.

e Tobacco-specific nitrosamines (sum of NNN, NNK, NAB and NAT).

f Benzo[a]pyrene.

### Extraction methods

The G, CPS, CDM and 2S3 samples were only tested with extractions using sterile, purified water; the CDM2 samples were tested using both water extractions and extractions with sterile, anhydrous dimethyl sulfoxide (DMSO) (Sigma-Aldrich, Poole, UK). DMSO was use as a second extraction agent for “CDM2”, because of the inclusion in this product of some experimental flavoring agents with limited water solubility.

Extracts were prepared using the following procedure at concentrations of 500 mg of tobacco product per ml, and conducted using sterile containers and solutions, in order to minimise contamination from external sources. Appropriate numbers of sachets of SWS were cut approximately in half, and both the contents and the sachets were weighed and mixed with appropriate volumes of water or DMSO to produce the required w/v concentration. If the tobacco was not finely divided then brief homogenisation was performed. Extractions were performed for 24 h at 37°C, with shaking. At the end of the 24 h extraction period, extracts were centrifuged at approximately 1800g for 30 min, and heavy particulates removed by decanting off the supernatant. The supernatant was then centrifuged at 25,000g for 30 min, and fine particulates removed by decanting off the supernatant. The final supernatant was adjusted to pH 7.4 ± 0.2 with hydrochloric acid or sodium hydroxide (water extracts only). The resulting extracts were filter-sterilized using a 0.2 μm pore size filter (pre-filtering using a larger pore size was performed where required). Where extracts were prepared in two or more separate flasks, extracts were pooled prior to use in the assay. Aliquots of extracts were stored at approximately −80°C, and used within 3 months of extraction. Since extracts of SWS and reference products were each prepared according to the above methodology, with the particulate matter removed prior to assay, stated concentrations for these extracts should be considered to be in terms of equivalent weight of test material, corresponding to the concentration of test material present during the extraction phase.

For the *Salmonella* assays, treatments were performed using additions of 0.5 ml water extract per plate or 0.1 ml of DMSO extract per plate. For the other assays, treatments were performed using 10% v/v additions of water extract and 1%, 1.5%, or 2% v/v additions of the DMSO extract.

### Nicotine contents

Nicotine concentrations were assessed using high-performance liquid chromatography; extraction blanks were used as negative controls in each assay system (Tambwekar et al., 2003). Three additional concentrations (200, 300 and 400 mg/ml) were used in addition to that used for the toxicology testing (500 mg/ml).

### *In vitro* assays

The four assays used were the *S. typhimurium* reverse-mutation assay, the MLA, an *in vitro* micronucleus assay (MNAvit), and the NRU assay.

### Ames assay

Extracts were assessed in a bacterial mutation assay system, using *S. typhimurium* tester strains TA98 (frame-shift), TA100 (base-pair substitution), TA102 (base-pair substitution), TA1535 (base-pair substitution) and TA1537 (frameshift) using pre-incubation methodology. Bacteria were originally obtained from the UK National Centre for Type Cultures and from Glaxo Group Research Ltd. Testing was performed in the absence and presence of S9 (Molecular Toxicology Inc., Boone, NC), as described in OECD guideline 471 (OECD, 1997a). Six concentrations were used for the water extracts: 0.08, 0.40, 2, 10, 50 and 250 mg per plate equivalent (OECD guideline 471 recommends a maximum test concentration for soluble non-cytotoxic substances of 5 mg/plate). Six concentrations were also used for the DMSO extract: 0.016, 0.08, 0.40, 2, 10 and 50 mg per plate equivalent. Three plates were used for each treatment and for the positive controls, with five for the solvent control.

The highest concentrations used were considered to be the highest achievable concentrations in this assay, using the highest extraction concentration that could practically be extracted from the test materials, and the largest volume additions that the assay system could tolerate for each solvent.

The following positive control substances (Sigma-Aldrich) and their concentrations were used for the different tester strains and S9 conditions: 2-nitrofluorene (2NF, TA98, −S9, 5 μg/plate), sodium azide (NaN_3_, TA100, TA1535, both −S9, 2 μg/plate), 9-aminoacridine (AAC, TA1537, −S9, 50 μg/plate), mitomycin C (MMC, TA102, −S9, 0.2 μg/plate), benzo[a]pyrene (B[a]P, TA98, ⊞S9, 10 μg/plate) and 2-aminoanthracene (AAC, TA100, TA1535, TA1537, and TA102, all ⊞S9, 5 μg/plate except for TA 102, 20 μg/plate).

### MLA assay

Extracts were assessed in duplicate cultures of mouse lymphoma L5178Y thymidine kinase (*tk^⊞/−^*) cells (a gift from Dr. D. Clive, Burroughs-Wellcome, NC), as described in OECD Guideline 476 (OECD, 1997c). Treatments were conducted using 3- and 24-h exposures in the absence of S9, and a 3-h treatment in the presence of S9 (Clive et al., 1995; Moore et al., 2006), using six concentrations for the water extractions (5,10, 20,30, 40 and 50 mg/ml equivalent) and eight concentrations for the DMSO extractions (7.5, 15, 30, 45, 60, 65, 70 and 75 mg/ml equivalent). Treatments were assessed for their effects on relative total growth (RTG) and mutant frequency (MF).

The positive controls (single plate, Sigma-Aldrich) used were 4-nitroquinoline 1-oxide (NQO, −S9 condition) and B[a]P (⊞S9 condition), at low and high concentrations of 0.05 and 0.1 μg/ml (NQO, 24 h), 0.15 and 0.20 μg/ml (NQO, 3 h), and 2 and 3 μg/ml (B[a]P, both time points), respectively.

### MNAvit assay

Extracts were assessed in duplicate cultures of Chinese hamster fibroblast V79 cells (a gift from Dr. E Massey, BAT, UK) in an *in vitro* MNA, according to OECD guidelines 474 (OECD, 1997b) and 487 (OECD, 2007). Four plates were used for the solvent controls and two plates for the positive controls. The target number of cells to be examined was 1000 for each plate. Treatments were conducted using 3 h (pulse) treatments in the absence and presence of S9, followed by a 17-h recovery period (3 ⊞ 17), and 20 h (continuous) treatment in the absence of S9. At least three concentrations were tested in addition to solvent controls, in the range 6.7-50 mg/ml for the water extracts and 0.27-7.5 mg/ml for the DMSO extracts.

The MNA assay detects clastogens and aneugens in cells that have undergone cell division during or after exposure to the test substance (Lorge et al., 2006). The assay typically uses protocols with and without the actin polymerization inhibitor cytochalasin B (a mycotoxin); however, we did not perform any testing without cytochalasin B. Addition of this compound prior to the targeted mitosis allows for the identification and selective micronucleus frequency in cells that have completed a single mitosis, because such cells are binucleate. Cytochalasin B (Sigma-Aldrich) inhibits cytokinesis (cell division), but not karyokinesis (nuclear division), resulting in the formation of binucleate cells. Thus, when micronuclei are only counted in binucleate cells, a true measurement of their induction can be obtained. Cytochalasin-B has been shown to have no effect on the overall assay (Lorge et al., 2006). Treatments were assessed for resulting counts of micro-nucleated binucleate (MNBN) cells.

The positive controls (Sigma-Aldrich) used were NQO (0.30 μg/ml) and vinblastin (VIN, 0.004 μg/ml), for the −S9 condition, and cyclophosphamide (CPA, 8 μg/ml) for the ⊞S9 condition.

### NRU assay

Extracts were assessed in Balb/c 3T3 mouse fibroblast cells (Borenfreund and Puerner, 1985; Babich and Borenfreund, 1990), purchased from the European Cell and Culture Collection. Cytotoxicity was measured by assessment of NRU after 24 h of exposure, expressed as the per cent cell survival. The eight concentrations were 0.39, 0.78, 1.56, 3.13, 6.25, 12.5, 25 and 50 mg/ml for the water extracts and 0.04, 0.08, 0.16, 0.31, 0.63, 1.25, 2.5 and 5 mg/ml for the DMSO extracts. Six replicates were performed for each concentration. The positive control used was sodium dodecyl sulfate (Sigma-Aldrich), used at a single concentration of 100 μg/ml.

Two experiments were performed: one compared samples “G”, “CPS” and “2S3”, and the other compared samples “CDM”, “CDM2” (water extraction), and “CDM2” (DMSO extraction).

### Endpoint analyses

For the *S. typhimurium* analyses, we used pairwise comparisons of mean revertant counts with the solvent control, using Dunnett's test and a significance level of *P <* 0.01. Dunnett's test makes adjustments for multiple corrections: it ensures that the type 1 error rate will be fixed at the desired level by incorporating correction factors into the design of the test table. The significance level used here (<0.01 rather than <0.05 used elsewhere) is well-established and is widely used for bacterial mutation assay data. An estimate was made of dose response, considered to be present if the two highest concentrations both produced significant differences from the solvent controls. We also considered the twofold rule (Cariello and Piegorsch, 1996) to infer positive results, modified to a threefold rule for tester strains TA1535 and TA1537 because of their relatively low number of background revertants (c. 10-20 per plate).

For the MLA we used pairwise comparisons of mean MF values with those in the solvent controls, using Dunnett's test and a significance level of *P <* 0.05. Linear trends in MF values were evaluated using χ^2^-tests and a significance level of *P <* 0.05. We also considered mean MF values that were at least 126 revertants per million cells greater than the mean MF in the solvent controls (termed the global evaluation factor) (Moore et al., 2006).

In the MNAvit study, a binomial dispersion test was used to determine homogeneity between replicates. This was followed by the use of Fisher's exact test and a significance level of *P <* 0.05 to compare numbers of MNBN cells in treated groups with those in the concomitant solvent control group. We also considered whether the results obtained for percentages of MNBN cells were higher than those seen in the historical solvent control. These historical values (from 53 prior studies) were 2.9% (3 ⊞ 17, −S9), 2.8% (3 ⊞ 17, ⊞ S9), and 3.0% (0 ⊞ 20, −S9).

For the NRU assay, pairwise comparisons of cell survival rates were made at the highest extract concentration, using SigmaPlot version 11.2 (San Jose, CA) and a significance level of *P <* 0.05.

## Results

### Nicotine determination

Excellent consistency in nicotine concentrations was obtained between separate water extracts, indicating that the extraction methodology provided consistent levels of extracted material. Estimated nicotine recovery levels (based on information provided on nicotine levels of the different materials used) from 200, 300, 400 and 500 mg/ ml equivalent water extracts were 84%, 78%, 76% and 73%, indicating that nicotine content does continue to increase proportionately over these increasing extraction concentrations, with only a relatively small reduction in recovery efficiency.

### Ames assay

Results of the *S. typhimurium* mutagenicity assays for sample “G” are given as Table 2, with separate listings for the −S9 and ⊞S9 conditions. Results for the other samples are provided in the supplementary material.

**Table 2:** Results of the *Salmonella mutagenesis* assay with water extracts of Swedish snus sample “G” (-S9 and ⊞9 conditions).

Treatment (mg/ml)		TA98	TA100	TA1535	TA1537	TA102
0	−S9	17 ± 6	108 ± 4	11 ± 3	14 ± 3	288 ± 13
0.08	−S9	ND	93 ± 11	24 ± 7*	13 ± 3	311 ± 6
0.4	−S9	33 ± 3*	89 ± 10	19 ± 5	16 ± 5	313 ± 6
2	−S9	23 ± 2	97 ± 8	13 ± 3	16 ± 2	308 ± 23
10	−S9	17 ± 1	91 ± 13	16 ± 5	16 ± 6	313 ± 8
50	−S9	19 ± 5	83 ± 7	21 ± 8	18 ± 5	338 ± 15*
250	−S9	21 ± 6	122 ± 9	45 ± 10*	26 ± 6*	390 ± 30*
Positive control	−S9	940 ± 210*	940 ± 84*	573 ± 22*	68 ± 9*	1573 ± 38*
0	⊞S9	27 ± 6	113 ± 6	20 ± 9	20 ± 4	262 ± 47
0.08	⊞S9	28 ± 5	82 ± 19	18 ± 4	17 ± 5	189 ± 75
0.4	⊞S9	27 ± 3	99 ± 6	17 ± 6	22 ± 4	200 ± 33
2	⊞S9	26 ± 2	92 ± 18	16 ± 1	15 ± 6	218 ± 86
10	⊞S9	29 ± 9	108 ± 14	15 ± 8	19 ± 6	216 ± 89
50	⊞S9	23 ± 10	79 ± 8	13 ± 5	19 ± 9	306 ± 24
250	⊞S9	43 ± 8	126 ± 12	43 ± 8*	40 ± 3*	323 ± 32
Positive control	⊞S9	249 ± 56*	1159 ± 99*	185 ± 112*	74 ± 57*	1016 ± 170*

Data expressed as mean (±SD) revertant colonies per plate. Five replicates were used for the solvent control; three replicates were used for all other treatments. Bold type indicates a doubling (TA98, TA100, TA102) or a tripling (TA1535, TA1537) of the mean revertant count for the solvent control. Positive controls and the doses used are described in the text.

* Significantly different (P < 0.01) from the solvent control, using Dunnett's test.

ND, no data obtained.

For both conditions, there were several mean rever-tant counts that reached statistical significance when compared with the solvent control; these counts were often limited to the highest concentration (i.e. there was little or no evidence for a dose-response relationship). There were three cases where the two highest concentrations were both significantly greater than the solvent control: sample “G” for TA102 in the −S9 condition (Table 2), sample “CDM” for TA1537 in the −S9 condition, and sample “CDM2” in the water extraction, TA102, −S9 condition. There were only two cases of a doubling (or a tripling) of the mean value for the solvent control, but these were not the same two cases as those described above. The cases here were sample “G”, TA1535, −S9 (mean of 45 revertants compared to 11, a factor of 4.1, Table 2), and sample “CPS”, TA1535, −S9 (mean of 43 revertants compared to 11, a factor of 3.9). The two different assessment methods thus provided different indications of possible mutagenic activity; both of these indications were effectively limited to the highest concentration being tested.

With one exception, mean revertant counts for the positive controls were in the range of 5-to-70-fold significant increases over the mean for the solvent control for the −S9 condition and 4-to-9-fold for the ⊞S9 condition. The exception was for the DMSO extractions of sample “CDM2” with strain TA1537: for the ⊞S9 condition the mean value for the positive control was 2.9 times higher than that for the solvent control.

### MLA assay

The results of the MLA for sample “G” are given as Table 3, with separate listings for the 3-h treatments (both S9 conditions) and the 24-h treatments (-S9 only). Results for the other samples are provided in the supplementary material.

**Table 3:** Results of the mouse lymphoma assay with water extracts of Swedish snus sample “G” (3-h and 24-h treatment).

Treatment (mg/ml)		%RTG	MF		%RTG	MF
0 (3-h)	−S9	100	112	⊞S9	100	74.7
5	−S9	105	104	⊞S9	108	69.2
10	−S9	125	86.1	⊞S9	101	72.5
20	−S9	134	78.8	⊞S9	113	72.1
30	−S9	136	75.3	⊞S9	97	88.7
40	−S9	101	91.8	⊞S9	106	54.0
50	−S9	107	89.9	⊞S9	88	97.0
Positive control-L	−S9	62	728*	⊞S9	57	552*
Positive control-H	−S9	83	533*	⊞S9	30	992*
0 (24-h)	−S9	100	147†			
10	−S9	95	144			
20	−S9	71	184			
30	−S9	67	177			
40	−S9	69	186			
45	−S9	54	177			
50	−S9	42	223*			
Positive control-L	−S9	67	288*			
Positive control-H	−S9	48	431*			

RTG, Relative total growth; MF, Mutant frequency, the number of revertant cells x 10^−6^ viable cells 2 days after treatment.

Two replicates were used for each treatment, with only a single plate for the positive controls. Bold type indicates that the global evaluation factor (a mutant frequency of 126 × 10 ^−6^ revertant cells greater than the mutant frequency in the solvent control) was exceeded. Positive controls were 4-nitroquinoline-1-oxide for the −S9 condition and benzo[a]pyrene for the ⊞S9 condition. See text for doses used.

* Significantly different (P < 0.05) from the solvent control, using Dunnett's test.

† A significant (P < 0.05) linear trend, using the χ^2^-test.

There were several mean MFs that reached statistical significance when compared with the solvent control; these differences were sporadic with no apparent dose-response relationship. There were two clear exceptions, both in the 24-h −S9 condition. The first was sample “CDM”, where the two highest treatments both had mean MFs that were significantly greater than the mean for the solvent control. The second was sample “CDM2”, water extraction, where the three highest treatments all had mean MFs that were significantly greater than the mean for the solvent control. Significant linear trends in MFs were noted for several of the 3-h ⊞S9 and 24-h −S9 conditions; none were observed for the 3-h −S9 condition.

There were two cases of mean MFs greater by at least 126 revertants per million cells than the mean for the solvent control, and these were for the same samples as those noted above for conventional statistical significance. The highest treatments for the 24-h-S9 condition with samples “CDM” and “CDM2”, water extraction, had mean MFs of 270 and 327, respectively, compared with the mean value for the solvent control group of 103.

The two positive responses described above were both associated with considerable toxicity: the RTGs for both at the highest concentration were only 8%, less than the suggested minimum of 10% (Moore et al., 2006).

Mean MFs for the positive controls (both doses) were in the range of 2-to-17-fold significant increases over the mean value for the solvent control and were considerably greater than the global evaluation factor.

### MNAvit assay

Homogeneity testing did not reveal any major discrepancies between replicates.

The results of the MNAvit assay for sample “G” are given as Table 4, with separate listings for the 3⊞17 h treatments (both of the S9 conditions) and the 20 h treatment (the −S9 condition only). Results for the other samples are given in the supplementary material.

**Table 4:** Results of the in vitro micronucleus assay with water extracts of Swedish snus sample “G”.

Dosing regimen (hours treatment ⊞ hours recovery, S9 status)								
3 ⊞ 17, −S9			3 ⊞ 17, ⊞S9			20 ⊞ 0,-S9		
Dose	MNBN		Dose	MNBN		Dose	MNBN	
mg/ml	Cells	%	mg/ml	Cells	%	mg/ml	Cells	%
0	34	0.85	0	71	0.89	0	45	1.13
16.4	16	0.8	8.39	47	1.18	6.71	24	1.2
32	25	1.25	25.6	40	1	8.39	24	1.2
50	20	1	50	72	1.80*	10.5	27	1.35
NQO	35	2.42*	CPA	235	5.88*	VIN	30	55.6*
						NQO	174	9.23*

MNBN, micro-nucleated binucleate cells; NQO, 4-nitroquinoline 1-oxide; CPA, cyclophosphamide; VIN, vinblastin. See text for doses used.

Two replicates were used for each treatment and for the positive controls; four replicates were used for the solvent controls. The target was 2000 binucleate cells per replicate. Bold type indicates values that exceeded the historical range for the solvent control (see text).

* Significantly different (P < 0.05) from the solvent control, as assessed by Fisher's exact test. ⊞excessive cytotoxicity.

There were several incidences of MNBN incidences that reached statistical significance when compared with the concomitant solvent control; in only two cases did the incidences exceed the historical range for the solvent control. The first of these cases was for sample “CDM2”, water extraction, in the 3 ⊞ 17 h, −S9 condition. At the highest concentration tested the mean MNBN response was 4.1%, compared with 0.85% for the concomitant solvent controls and with 2.9% for historic solvent controls. However, there was considerable cytotoxicity (70%) associated with this response. The second case was the 2S3 reference product, in the 3 ⊞ 17 h, ⊞S9 condition. At the highest concentration tested the mean MNBN response here was 4.08%, compared with 0.89% for the concomitant solvent controls and with 2.8% for historic solvent controls. Cytotoxicity was not excessive in this case (22%), and so the 2S3 response is unequivocally positive.

Positive controls produced responses that were significantly increased when compared with concomitant solvent controls. With one exception, the positive control responses were also significantly increased when compared with historical solvent controls. The exception was NQO in the 3 ⊞ 17 h, −S9 condition, where the mean value (2.42%) was less than the historical mean value, even though it was almost threefold higher than the concomitant solvent control (comparable to the positive control for the TA1537 ⊞S9 condition of the Ames assay). When used at the same dose in the 20 ⊞ 0 h, −S9 condition, NQO produced an approximately eightfold increase over concomitant solvent controls and an approximately threefold increase over historical solvent controls.

### NRU assay

**Figure 1:**
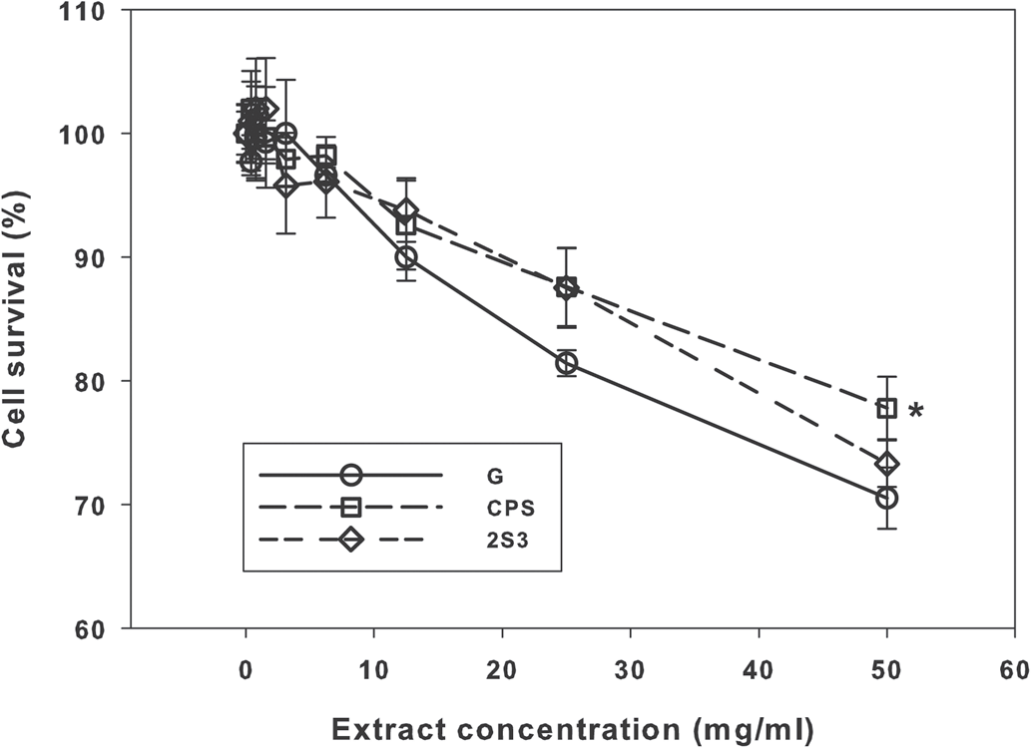
Results of the neutral red uptake assay with samples “G”, “CPS” and “2S3”. Means ± SD *(n =* 6). ‘Significantly different *(P <* 0.05) from the other two means.

**Figure 2:**
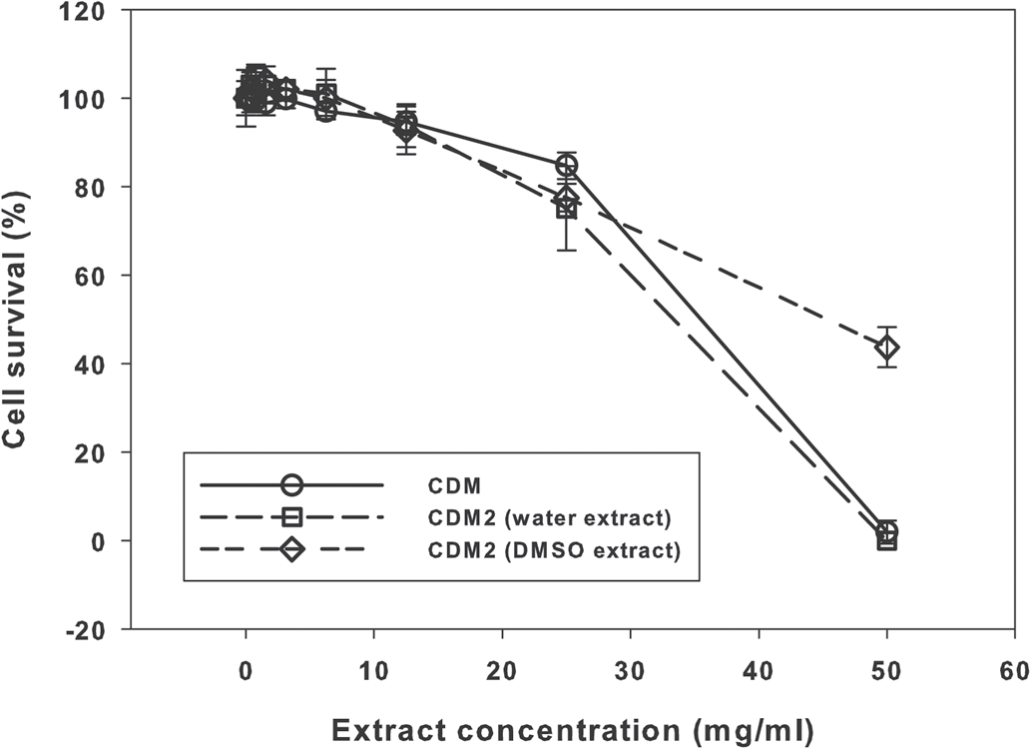
Results of the neutral red uptake assay with samples “CDM” and “CDM2”. Means ± SD (n *=* 6).

Results of the two experiments are presented as Figures 1 and 2.

The increasing cytotoxicity (decreasing cell survival) of the extracts from samples “G”, “CPS” and “2S3” were very similar, with the lowest attained survival being approximately 75% (Figure 1). At the highest extract concentration, the mean cell survival for the “CPS” sample was significantly greater (by about 10%) than those for the “G” and “2S3” samples (Holm-Sidak test, *P <* 0.05, Figure 1).

The decline in cell survival in the “CDM” and “CDM2” (water extraction) samples with increasing extract concentration was effectively identical (Figure 2), with zero survival at the highest concentration. However, cell survival for the “CDM2” sample (DMSO extraction) at the highest concentration was 44% (Figure 2).

There was minimal cell survival with the positive control.

## Discussion

We showed that various types of *in vitro* toxicology testing, including those tests classically used to predict carci-nogenicity in humans, are feasible for STP such as SWS. Positive control substances demonstrated that each of the tests was working correctly.

We also showed that for SWS in assays such as *S. typhimurium,* mouse lymphoma and *in vitro* micronucleus, the results provided no clear biologically-relevant positive responses, as opposed to the statement of “poor performance” made by others (Johnson et al., 2009). In most cases the results for the reference moist snuff were also negative, but there was one unequivocally positive response. Using carefully controlled assays with positive controls, we showed that cytotoxicity can be controlled and indeed (with some exceptions) this was not a problem in the work we describe here. The broadly negative data presented here for SWS contrast strongly with data for cigarettes where the tobacco is combusted; for these products, substantial genotoxicity has been demonstrated repeatedly (DeMarini et al., 2008).

Normally, genetic toxicology data such as those we present here would be the first step in a toxicologi-cal evaluation that would end with epidemiology. The reverse is the case here, with extensive epidemiology but minimal genetic toxicology. Our work therefore represents a unique case of being able to “look back” on the toxicological evaluation process; it provides validation of the genetic testing of non-combusted tobacco products. Cigarette smoke on the other hand has shown strong evidence of carcinogenicity by epidemiology and positive results from genetic toxicology tests.

A limitation of the work described here is the fact that a full set of chemical analyses was not made on the actual products tested (nicotine only was analyzed). However, a full set of analyses was made on identical products, and these data are presented in Table 1. Another limitation is the lack of agreement in the literature on the statistical criteria to be used to determine a positive response, particularly for the *Salmonella* mutagenicity assay where the OECD guidelines simply state that “there are several criteria for determining a positive result.” A further limitation is our exclusive use of aqueous extracts, supplemented in one case with DMSO extracts. We are aware of published work with extractions using artificial saliva, and the use of alternative extraction agents may be an avenue for future work.

These broadly negative findings in a controlled laboratory setting add to the large amount of epide-miological data from Scandinavia (Colilla, 2010; Lee, 2011), data showing that SWS are associated with considerably lower carcinogenic potential when compared with tobacco products involving combustion of the tobacco (US Department of Health and Human Services, 2004).

## Conclusions

For each of the assays, data obtained for negative and positive controls confirmed that the test systems were working correctly.

### Ames assay

Extracts of the four SWS and the 2S3 reference did not induce clear increases in MF in five strains of *S. typhimurium,* both in the absence and in the presence of S9. Two different methods for evaluating the data produced conflicting results. The observed increases in revertant numbers were limited to extreme concentrations of the test material (well in excess of concentrations required by regulatory guidelines, such as OECD 471), and so these findings are considered to be of limited relevance to an overall risk assessment for SWS.

### MLA assay

Extracts of the four SWS and the 2S3 reference did not induce clear increases in MF in L5178Y *tk^⊞/−^* cells, both in the absence and in the presence of S9. For the 3-h treatments, no increase in MFs over the global evaluation factor was noted. In two cases with the 24-h treatments, MFs were greater than the global evaluation factor. In each of these cases the RTG values were substantially reduced (indicating considerable cytotoxicity), so these findings are considered to be of limited relevance to an overall risk assessment for SWS.

### MNAvit assay

Extracts of the four SWS did not show increased MNBN counts in Chinese hamster fibroblast cells, with an unequivocally positive result for the 2S3 reference at the highest dose in the 3 ⊞ 17 h ⊞S9 condition.

### NRU assay

No obvious differences were noted between the cytotoxicity results noted in Balb/c 3T3 mouse fibroblast cells for the “G”, “CPS” and “2S3” samples, with the highest extract concentrations only producing approximately 75% cytotoxicity. Complete cytotoxicity was seen with the “CDM” and “CDM2” samples at the highest concentration, with some apparent reduction in cytotoxicity in the “CDM2” sample when DMSO extraction was used.
